# Supplementary material for: Symmetry-protected metallic and topological phases in penta-materials
Source: Sci Rep. 2019 Sep 4;9:12754. doi: 10.1038/s41598-019-49187-w (PMC6726764; doi:10.1038/s41598-019-49187-w)
Supplement: Supplementary file 1 — Supplementary information to ”Symmetry-protected metallic and topological phases in penta-materials” [file 41598_2019_49187_MOESM1_ESM.pdf]

Supplementary information to ”Symmetry-protected metallic and topological phases in penta-materials”

Sergio Bravo[1], Julián Correa[2], Leonor Chico[3], and Mónica Pacheco[1,*]

[1]Universidad Técnica Federico Santa María, Departamento de Física, Valparaíso, Casilla 110-V, Chile.
[2]Universidad de Medellín, Facultad de Ciencias Básicas, Medellín, Colombia
[3]Materials Science Factory, Instituto de Ciencia de Materiales de Madrid, Consejo Superior de Investigaciones Científicas, C/ Sor Juana Inés de la Cruz 3, 28049 Madrid, Spain
[*]monica.pacheco@usm.cl

ABSTRACT

In this supplementary information we include the character tables for the double groups of the wavevector at different points and lines of the Brillouin zone. The character information and the notation for the symmetry operations is adapted from the Bilbao crystallographic server.

Character tables

	I	2_{001}	$\overline{4}^{+}_{001}$	$(2_{010}\|\frac{1}{2}\frac{1}{2}0)$	$(m_{110}\|\frac{1}{2}\frac{1}{2}0)$	I^d	$\overline{4}^{+d}_{001}$
Γ_1	1	1	1	1	1	1	1
Γ_2	1	1	-1	1	-1	1	-1
Γ_3	1	1	-1	-1	1	1	-1
Γ_4	1	1	1	-1	-1	1	1
Γ_5	2	-2	0	0	0	2	0
$\bar{\Gamma}_6$	2	0	-$\sqrt{2}$	0	0	-2	$\sqrt{2}$
$\bar{\Gamma}_7$	2	0	$\sqrt{2}$	0	0	-2	-$\sqrt{2}$

Table 1. Character table for double space group no. 113 and Γ point.

	I	2_{001}	$(2_{010}\|\frac{1}{2}\frac{1}{2}0)$	$(2_{100}\|\frac{1}{2}\frac{1}{2}0)$	I^d	2^d_{001}	$(2^d_{010}\|\frac{1}{2}\frac{1}{2}0)$	$(2^d_{100}\|\frac{1}{2}\frac{1}{2}0)$
X_1	2	0	0	0	2	0	0	0
$\bar{X}_2$	1	-i	1	-i	-1	i	-1	i
$\bar{X}_3$	1	i	-1	-i	-1	-i	1	i
$\bar{X}_4$	1	-i	-1	i	-1	i	1	-i
$\bar{X}_5$	1	i	1	i	-1	-i	-1	-i

Table 2. Character table for the double group at X point.

	I	2_{001}	I^d	$(2^d_{100}\vert\frac{1}{2}\frac{1}{2}0)$
Y_1	1	$e^{i\pi u}$	1	$e^{i\pi u}$
Y_2	1	$e^{i\pi(1+u)}$	1	$e^{i\pi(1+u)}$
$\bar{Y}_3$	1	$e^{-i\pi(\frac{1}{2}-u)}$	-1	$e^{i\pi(\frac{1}{2}+u)}$
$\bar{Y}_4$	1	$e^{i\pi(\frac{1}{2}+u)}$	-1	$e^{-i\pi(\frac{1}{2}-u)}$

Table 3. Character table for the double group at Y line. Where $k_Y = (\frac{1}{2}, u, 0)$ with $u \in (0, \frac{1}{2})$.

	I	2_{001}	$\overline{4}^+_{001}$	$\overline{4}^-_{001}$	$(2_{010}\vert\frac{1}{2}\frac{1}{2}0)$	$(2_{100}\vert\frac{1}{2}\frac{1}{2}0)$	$(m_{110}\vert\frac{1}{2}\frac{1}{2}0)$	$(m_{1\bar{1}0}\vert\frac{1}{2}\frac{1}{2}0)$
M_1	1	-1	i	$-i$	i	$-i$	1	-1
M_2	1	-1	$-i$	i	i	$-i$	-1	1
M_3	1	-1	$-i$	i	$-i$	i	1	-1
M_4	1	-1	i	$-i$	$-i$	i	-1	1
M_5	2	2	0	0	0	0	0	0
$\bar{M}_6$	2	0	$\sqrt{2}i$	$-\sqrt{2}i$	0	0	0	0
$\bar{M}_7$	2	0	$-\sqrt{2}i$	$\sqrt{2}i$	0	0	0	0

Table 4. Character table for the double group at M point.

	I^d	2^d_{001}	$\overline{4}^{+d}_{001}$	$\overline{4}^{-d}_{001}$	$(2^d_{010}\vert\frac{1}{2}\frac{1}{2}0)$	$(2^d_{100}\vert\frac{1}{2}\frac{1}{2}0)$	$(m^d_{110}\vert\frac{1}{2}\frac{1}{2}0)$	$(m^d_{1\bar{1}0}\vert\frac{1}{2}\frac{1}{2}0)$
M_1	1	-1	i	$-i$	$-i$	i	1	-1
M_2	1	-1	$-i$	i	$-i$	i	-1	1
M_3	1	-1	$-i$	i	i	$-i$	1	-1
M_4	1	-1	i	$-i$	i	$-i$	-1	1
M_5	2	2	0	0	0	0	0	0
$\bar{M}_6$	-2	0	$-\sqrt{2}i$	$\sqrt{2}i$	0	0	0	0
$\bar{M}_7$	-2	0	$\sqrt{2}i$	$-\sqrt{2}i$	0	0	0	0

Table 5. Character table for the double group at M point (continuation).
